# Alterations of local spontaneous brain activity and connectivity in adults with high-functioning autism spectrum disorder

**DOI:** 10.1186/s13229-015-0026-z

**Published:** 2015-05-24

**Authors:** Takashi Itahashi, Takashi Yamada, Hiromi Watanabe, Motoaki Nakamura, Haruhisa Ohta, Chieko Kanai, Akira Iwanami, Nobumasa Kato, Ryu-ichiro Hashimoto

**Affiliations:** Medical Institute of Developmental Disabilities Research, Showa University, 6-11-11 Kita-karasuyama, Setagaya-ku, Tokyo Japan; Department of Psychiatry, School of Medicine, Showa University, 6-11-11 Kita-karasuyama, Setagaya-ku, Tokyo Japan; ATR Brain Information Communication Research Laboratory Group, Kyoto, Japan; Kanagawa Psychiatric Center, Kinko Hospital, 2-5-1 Serigaya, Yokohama, Kanagawa Japan; Department of Language Sciences, Graduate School of Humanities, Tokyo Metropolitan University, 1-1 Minami-Osawa, Hachioji-shi, Tokyo Japan

**Keywords:** Autism spectrum disorder, Resting-state functional magnetic resonance imaging, Spontaneous activity, Local connectivity, Amplitude of low-frequency fluctuation

## Abstract

**Background:**

Previous autism research has hypothesized that abnormalities of functional connectivity in autism spectrum disorder (ASD) may vary with the spatial distance between two brain regions. Although several resting-state functional magnetic resonance imaging (rsfMRI) studies have extensively examined long-range (or distant) connectivity in the adult ASD brain, short-range (or local) connectivity has been investigated in less depth. Furthermore, the possible relationship between functional connectivity and brain activity level during the resting state remains unclear.

**Methods:**

We acquired rsfMRI data from 50 adults with high-functioning ASD and 50 matched controls to examine the properties of spontaneous brain activity using measures of local and distant connectivity together with a measure of the amplitude of brain activity, known as fractional amplitude of low-frequency fluctuation (fALFF). The two connectivity measures were calculated using a common graph-theoretic framework. We also examined the spatial overlaps between these measures and possible relationships of these disrupted functional measures with autistic traits assessed by the Autism-Spectrum Quotient (AQ).

**Results:**

Compared to the controls, participants with ASD exhibited local over-connectivity in the right superior frontal gyrus and middle frontal gyrus, accompanied by local under-connectivity in the bilateral fusiform gyri (FG) and right middle temporal gyrus (MTG). On the other hand, we did not find any significant alterations in distant connectivity. Participants with ASD also exhibited reduced fALFF in the right middle occipital gyrus, lingual gyrus, and FG. Further conjunction and spatial overlap analyses confirmed that the spatial pattern of reduced fALFF substantially overlapped with that of local under-connectivity, demonstrating the co-occurrence of disrupted connectivity and spontaneous activity level in the right inferior occipital gyrus, posterior MTG (pMTG), and FG. Finally, within the ASD group, disrupted local connectivity in the right pMTG significantly correlated with the “social interaction” subscale score of the AQ.

**Conclusions:**

These findings revealed local functional disruptions in the occipital and temporal regions, especially the right FG and pMTG, in the form of co-occurrence of spontaneous brain activity level and local connectivity, which may underline social and communicative dysfunctions in adult ASD.

**Electronic supplementary material:**

The online version of this article (doi:10.1186/s13229-015-0026-z) contains supplementary material, which is available to authorized users.

## Background

Autism spectrum disorder (ASD) has been widely recognized as a complex neurodevelopmental disorder characterized by atypical anatomical and functional connectivity between brain regions [1]. However, there is not enough consensus on what kind of abnormality that characterizes the neurobiological underpinnings of this disorder, partly because of multiple confounding factors that may impact on connectivity, including developmental stage [2, 3] and cognitive state [4]. Previous functional magnetic resonance imaging (fMRI) studies have proposed that the ASD brain may be characterized by under-connectivity [5, 6]. Although the under-connectivity theory of ASD has been supported by many fMRI studies [5, 7–10], some of the recent fMRI studies have also provided evidence for over-connectivity [11–14]. In the face of contradictory findings, current research has led to the proposal of a disrupted connectivity theory that includes both under- and over-connectivity [15]. In this theory, the spatial distance of connectivity is considered a key factor determining the direction of alteration in functional connectivity such that the ASD brain may be characterized by reduced distant connectivity and enhanced local connectivity.

To date, a number of fMRI studies on ASD have reported atypical connectivity between relatively distant brain areas [16]. In contrast, the number of fMRI studies investigating local connectivity is limited, which may be partly due to uncertainty in the definition of “local” connectivity. Originally, local connectivity has been characterized at the mesoscopic level of the brain. Based on evidence of *postmortem* studies [17], Courchesne and Pierce [18] hypothesized that a series of pathological processes involving neuroinflammation, migration defects, and defective apoptosis might led to malformation of minicolumn circuitry particularly in the frontal cortex. In addition to the mesoscopic evidence, a few fMRI studies have investigated local connectivity in the ASD brain using regional homogeneity (ReHo) that quantifies, by Kendall’s coefficient of concordance (KCC), the similarity in the time-series data between a focal voxel and its direct neighbors (e.g., 6, 18, or 26 voxels) [19]. As the first fMRI study in ASD, Paakki et al. [20] examined the time-series of resting-state fMRI (rsfMRI) in adolescents with ASD and calculated the KCC from 26 nearest neighbor voxels; they found that adolescents with ASD exhibited increases of ReHo in the right thalamus and left inferior frontal gyrus, together with significant reductions in the right middle temporal gyrus (MTG) and superior temporal gyrus (STG). Similarly, Shukla et al. [21] examined this measure using a different spatial scale (six voxels) from task-based fMRI data. After regressing out the task effect, they observed that individuals with ASD showed decreases of ReHo in the bilateral middle frontal gyri (MFG) and superior frontal gyri (SFG) accompanied with increases in the right MTG and left amygdala. Although these two studies on child and adolescent ASD identified patterns of both over- and under-connectivity at the local scale, the spatial patterns of the two types of alterations did not match between the studies.

Recently, Sepulcre et al. [22] proposed a method for simultaneously quantifying both local and distant connectivity based on a graph-theoretic framework. In this approach, a functional brain network consists of a set of nodes (voxels) and edges (functional connectivity among nodes), and the network is embedded in the anatomical space in order to consider the Euclidean (or spatial) distance among nodes. Local and distant connectivity of a node (a single voxel) are then computed as the number of connections inside (local) or outside (distant) of the radius sphere centered on that focal node. This approach may allow more robust evaluation of local connectivity than ReHo, because local connectivity is the summation of connections from a larger number of voxels to a focal node and therefore may alleviate the effects of artifacts.

Maximo et al. [23] recently examined local connectivity by applying the graph-theoretic framework proposed by Sepulcre et al. [22] to the analysis of spontaneous brain activity in adolescents with ASD. They also examined ReHo, and both of the measures were calculated with multiple spatial scales (local connectivity 6- and 14-mm radii; ReHo 7, 19, 27, and 407 voxels). They demonstrated that the coarse spatial scale (>6-mm radius) yielded a more stable estimation for local connectivity. Furthermore, they found evidence of atypical local connectivity in adolescents with ASD in which local under-connectivity was identified in the bilateral posterior cingulate cortices (PCC), accompanied by local over-connectivity in the right MFG. Although there have been several other studies that have examined local connectivity in adolescents with ASD [4, 20, 21, 23, 24], to date, no studies have investigated local connectivity of adults with ASD.

While previous rsfMRI studies on neurotypical populations have mainly examined the synchronicity of spontaneous activity among brain regions, some rsfMRI studies have also investigated the magnitude of low-frequency oscillations of brain activity using a measure called the amplitude of low-frequency fluctuation (ALFF) or fractional ALFF (fALFF) [25]. In spite of potential implications of the measure with disease states of various brain disorders [26–29], to the best of our knowledge, only two recent fMRI studies on ASD have analyzed the amplitude of low-frequency fluctuation of spontaneous brain activity. Di Martino et al. [30] demonstrated increases of fALFF in the right MFG and reductions of fALFF in the left occipital pole using a large multi-site dataset. On the other hand, Supekar et al. [31] revealed that, compared to typical controls, children with ASD showed increases of the mean global functional connectivity, accompanied by increased mean ALFF. These previous studies have reported multiple forms of manifestations of atypical spontaneous brain activity in ASD. However, such findings have not yet led to an integrative understanding, partly because each measure has been examined largely in isolation or under a different analytical framework, which makes comparisons among multiple measures difficult.

In the present study, we aimed to achieve two major goals to advance our understanding of the abnormalities in spontaneous brain activity in adults with ASD. First, we aimed to estimate local and distant functional connectivity under the same graph-theoretic framework. Although a few previous studies of resting-state fMRI in adolescents with ASD have compared local connectivity under the same analytical framework [23, 24], no studies have applied this approach to adults with ASD. Applying this approach to the adult population is important considering the evidence of an altered developmental trajectory of functional connectivity from adolescence to adulthood in ASD [2, 32]. Second, we aimed to reveal possible associations between alterations in functional connectivity and those in the magnitude of brain activity. Commonalities of altered brain regions identified by different physiological measures can aid towards an integration of diverse findings. In relation to these two goals, we hypothesized that (1) adults with ASD would show distant under-connectivity and local under- and over-connectivity in several brain regions (e.g., the frontal, occipital, and temporal cortices); (2) altered fALFF in the ASD group would be found particularly in the occipital regions, given an observation of a previous finding using the same measure [30]; (3) atypical connectivity in some regions might co-occur with fALFF alterations; and (4) autistic traits would be associated with some of these measures of spontaneous brain activity in regions particularly related to social and communicative functions.

## Methods

### Participants

Fifty adults with ASD were recruited from outpatient units of the Karasuyama Hospital, Tokyo, Japan. A team of three experienced psychiatrists and a clinical psychologist assessed all patients. All patients were diagnosed with ASD based on the criteria of the Diagnostic and Statistical Manual of Mental Disorders, Fourth Edition (DSM-IV) and a medical chart review. The assessment consisted of participant interviews about developmental history, present illness, life history, and family history and was performed independently by a psychiatrist and a clinical psychologist in the team. Patients were also asked to bring suitable informants who had known them in early childhood. At the end of the interview, the patients were formally diagnosed with a pervasive developmental disorder by the psychiatrist if there was a consensus between the psychiatrist and clinical psychologist; this process required approximately 3 hours. A group of 50 age- and gender-matched normal controls (NCs) was recruited by advertisements and acquaintances. None of the NCs reported any severe medical problem or any neurological or psychiatric history. None of them satisfied the diagnostic criteria for any psychiatric disorder.

The intelligence quotient (IQ) scores of all participants with ASD were evaluated using either the Wechsler Adult Intelligence Scale Third Edition (WAIS-III) or the WAIS-Revised (WAIS-R), while those of NCs were estimated using a Japanese version of the National Adult Reading Test (JART) [33]. Although there are several minor changes in WAIS-III from WAIS-R (e.g., increase of items), the number of core items remained generally unchanged. Therefore, we considered that WAIS-R and –III were essentially the same with regard to measuring the full-scale IQ score of individuals with ASD. Regarding the JART, Matsuoka et al. [33] investigated the relationships between WAIS-R and JART in NCs and demonstrated that JART successfully predicted the full-scale IQ score, at least in the normal population. Every participant with ASD was considered to be high functioning, because his or her full-scale IQ score was higher than 80. Participants completed the Japanese version of the Autism-Spectrum Quotient (AQ) test [34]. To characterize more specific autistic traits of each participant, we computed the subscale scores of “social interaction” and “attention to detail,” using the method proposed by Hoekstra et al*.* [35]. Table 1 shows the demographic and clinical data of participants in both the ASD and NC groups. No demographic data (age, gender, and IQ) showed significant differences between the two groups (all *p* ≥ 0.3).

**Table 1:** The demographic data for the participants

	NC (male 43, female 7)			ASD (male 43, female 7)			Statistics	
	Mean	SD	Range	Mean	SD	Range	*df*	*P* value
Age (years)	31.60	7.60	19–49	30.82	7.39	19–50	98	0.60
Estimated IQ	108.09	8.98	87.46–119.80	105.60	14.12	82–134	98	0.30
AQ score	15.24	5.64	4–30	36.96	5.05	24–47	86	<0.001
AQ sub-scale								
Social interaction	81.42	12.34	53–106	126.5	12.23	101–150	86	<0.001
Attention to detail	21.87	3.93	15–37	26.14	4.38	17–35	86	<0.001

*NC* normal control, *ASD* autism spectrum disorder, *SD* standard deviation, *IQ* intelligence quotient, *AQ* autism-spectrum quotient

The Ethics Committee of the Faculty of Medicine of Showa University approved all of the procedures used in this study, including the method of obtaining consent, in accordance with the Declaration of Helsinki. Written informed consent was obtained from all the participants after fully explaining the purpose of this study. Any concern regarding the possibility of reduced capacity to consent on his or her own was not voiced by either the ethics committee or patients’ primary doctors.

### MRI data acquisition

All MRI data were acquired using a 1.5 T GE Signa system (General Electric, Milwaukee, WI, USA) with a phased-array whole-head coil. The functional images were acquired using a gradient echo-planar imaging sequence (in-plane resolution: 3.4375 × 3.4375 mm, echo time (TE) 40 ms, repetition time (TR) 2000 ms, flip angle 90°, slice thickness 4 mm with a 1-mm-slice gap, matrix size 64 × 64, 27 axial slices). Two hundred and eight volumes were acquired in a single run. The first four volumes were discarded to allow for T1 equilibration. During the scan, each of the participants was instructed to lie relaxed in the scanner, remain as still as possible, with his or her eyes closed, and yet to stay awake in the dim scanner room. In addition, a high-resolution T1-weighted spoiled gradient recalled (SPGR) 3D MRI image was acquired (in-plane resolution 0.9375 × 0.9375 mm, 1.4-mm-slice thickness, TR 25 ms, TE 9.2 ms, matrix size 256 × 256, 128 sagittal slices).

### rsfMRI preprocessing

All the rsfMRI data were processed mainly using the Statistical Parametric Mapping software (SPM 8) (Wellcome Department of Cognitive Neurology, London, UK), complemented with pinpoint uses of a part of Analysis of Functional NeuroImages (AFNI; http://afni.nimh.nih.gov/afni) [36] and FMRIB’s Software Library (FSL; http://fsl.fmrib.ox.ac.uk/fsl/fslwiki/) [37]. Unless otherwise specified, rsfMRI data were preprocessed using functions implemented in the SPM software. First, slice timing and head motion were corrected. No participant was excluded due to excessive motion (>±2 mm translation and >±2° rotation from the first volume in any axis). Next, rsfMRI data were skull-stripped using 3dAutomask and 3dcalc, and then time-series despiking was performed using 3dDespike implemented in the AFNI. We included time-series despiking in order to correct the abrupt signal changes in the analysis of fALFF, in which the condition of temporal continuity is required. Thus, the standard scrubbing method that removes corrupted time points cannot be applied. For each participant, a T1-weighted SPGR image was realigned along the mid-sagittal anterior-posterior commissure line and was segmented and reconstructed in order to create a skull-tripped T1-weighted image. The despiked fMRI data were co-registered to the skull-stripped T1-weighted image. The data were further spatially normalized to the standard Montreal Neurological Institute (MNI) template and resampled to a resolution of 2 × 2 × 2 mm. Finally, mean-based intensity normalization was performed to scale the mean of all of the voxels (over space and time) to a constant value (i.e., 10,000) with fslmaths implemented in FSL [25].

The removal of artifactual components from the time-series in each voxel was performed using the aCompCor method [38]. Briefly, this method identifies five principal components associated with physiological signals from the segmented white matter and cerebrospinal fluid regions, and then regresses out those time-series, together with those associated with 12 head motion parameters, consisting of 6 motion parameters and their first-order temporal derivatives, from the time-series in each voxel. Of note, the removal of global signal was not performed in order to avoid the risk of yielding spurious negative correlations [39].

The time-series were further preprocessed for the subsequent analyses. In this study, we included both motion regression and scrubbing, because several studies indicated that, although motion regression could remove a large portion of variance related to head motion from the rsfMRI data, some effects of head motion were retained after motion regression [40, 41]. In order to remove the effects of head motion on local connectivity as much as possible, we applied scrubbing as well. In this study, the scrubbing method was applied in the following order: (1) the frame-wise displacement (FD) and frame-by-frame signal intensity change (DVARS) were calculated immediately after head motion correction; (2) we regarded a volume as a motion-contaminated volume either when its FD value was greater than 0.5 mm or when its DVARS value was greater than the 75th percentile plus 1.5 times the interquartile range; (3) the signal values of the motion-contaminated volumes were interpolated by applying cubic spline function [42]; (4) for analyses of local and distant connectivity, a band-pass filter (0.009–0.08 Hz) was further applied in order to reduce the effects of low-frequency drifts and high-frequency physiological noises; (5) finally, the interpolated volumes were deleted. There were no significant differences between the two groups in the mean FD (NC 0.11±0.047 (mean±standard deviation); ASD 0.10±0.044; *t* = 1.57, *p* = 0.12) and DVARS (NC 1.29±0.21; ASD 1.28±0.17; *t* = 0.35, *p* = 0.73).

### Measures for spontaneous brain activity

#### Local and distant connectivity

Functional brain networks are composed of a set of nodes and edges. To construct functional brain networks, we regarded each voxel within a study-specific brain mask and functional connectivity between voxels as a node and edges, respectively. Following procedures described in previous studies [43, 44], we first generated a study-specific brain mask by including only voxels present in all 100 participants and those survived in a 40 % gray matter probability mask. The generated mask contained 146,594 voxels, each of which was regarded as a node in this study. For estimating the strength of functional connectivity between nodes, Pearson’s correlation coefficients between all possible pairs of nodes were calculated, which resulted in a 146,594 × 146,594 correlation matrix for each participant. A weighted, undirected graph was obtained by thresholding each element of correlation matrix at *r*_*ij*_ > 0.25 (*p* < 0.001), in accordance with a previous study [45].

Previous studies have used the weighted degree (or strength) of a weighted network to quantify the degree of connectivity of a focal node with the rest of nodes in the entire network regardless of physical distances between nodes [46, 47]. We distinguished between the local and distant connectivity by adopting a method described by Sepulcre et al*.* [22]. Local degree, or simply local connectivity, quantifies the degree of connectivity of a focal node with nodes inside of a pre-defined radius sphere. On the other hand, distant degree, or distant connectivity, quantifies the degree of connectivity of the node with nodes outside of the radius sphere. Since weighted graphs were constructed in this study, local and distant connectivity of the graph were characterized using local and distant strengths, instead of local and distant degrees. Following the criteria for determining the local and distant connectivity adopted by a previous study [48], local connectivity was computed as the weighted degree of connectivity within a 12-mm-radius sphere, while distant connectivity was calculated as the weighted degree of connectivity outside 25-mm-radius sphere.

#### Fractional amplitude of low-frequency fluctuation

For the assessment of the level of spontaneous brain activity, we generated an fALFF map in each participant using the REST toolbox (http://www.restfmri.net) [49]. After removal of artifactual components from the time-series of each voxel within the brain mask described before, we interpolated the motion-contaminated volumes (see “rsfMRI preprocessing” subsection) using a cubic spline function. In contrast to local and distant connectivity, we did not remove the interpolated volumes in order to maintain the temporal continuity. Then, we converted the time-series of each voxel into the frequency-domain using the fast Fourier transform and computed the power spectrum at each voxel. To be consistent with analyses of local and distant connectivity, a frequency range of interest was restricted to the range of from 0.009 to 0.08 Hz. The fALFF was computed as the ratio of the square root of the power spectrum averaged across that frequency range to that averaged across the entire detectable frequency range (0.00–0.25 Hz).

### Statistical analyses

#### Between-group comparisons

For group comparisons, a map of each measure was *z*-scored for each participant; each *z*-scored map was then spatially smoothed using a 6-mm full-width half-maximum Gaussian smoothing kernel [44]. Finally, statistical analyses were performed on each measure, while including age and the mean FD as nuisance covariates. In this study, multiple comparisons were corrected based on the Gaussian random field theory (*z* > 2.3; cluster-level significance *p* < 0.05, corrected).

#### Conjunction and spatial overlap analyses

It turned out that several brain regions showed significant group difference in local connectivity and fALFF. To examine whether there is any spatial overlap between alterations in these two measures, we conducted conjunction analyses and tested the conjunction null hypothesis [50]. Conjunction analyses were performed using a cluster-wise test with *z*-stats threshold of 2.3 (corrected-*p* threshold of 0.05).

Spatial overlaps between alterations in local connectivity and fALFF were further assessed using a Monte Carlo simulation with 5000 iterations, according to previous studies [51, 52]. At each iteration, we generated two whole-brain maps filled with values sampled from a Gaussian distribution. The two generated maps had the same smoothness as the two observed statistical maps. These simulated maps were thresholded using the same cluster-wise test with *z*-stats threshold of 2.3 (corrected-*p*-value threshold of 0.05), and then we counted the number of overlapped voxels. Finally, the probability of the number of overlapped voxels observed in the actual data was calculated using a null distribution of the number of overlapped voxels in the random setting.

#### Relationships between atypical measures and autistic traits

When significant between-group differences were found in any of the measures, three multiple linear regression analyses were performed to examine possible associations of each measure with the autistic traits assessed by the AQ (the total AQ score and the “social interaction” and “attention to detail” subscale scores) within the ASD group. Of note, age and the mean FD were included as nuisance covariates. In each measure, we generated a mask to perform statistical evaluations only on voxels showing significant between-group differences. Statistical significance was set at *p* < 0.05, false discovery rate (FDR) corrected for multiple comparisons.

## Results

### Local and distant connectivity

**Fig. 1:**
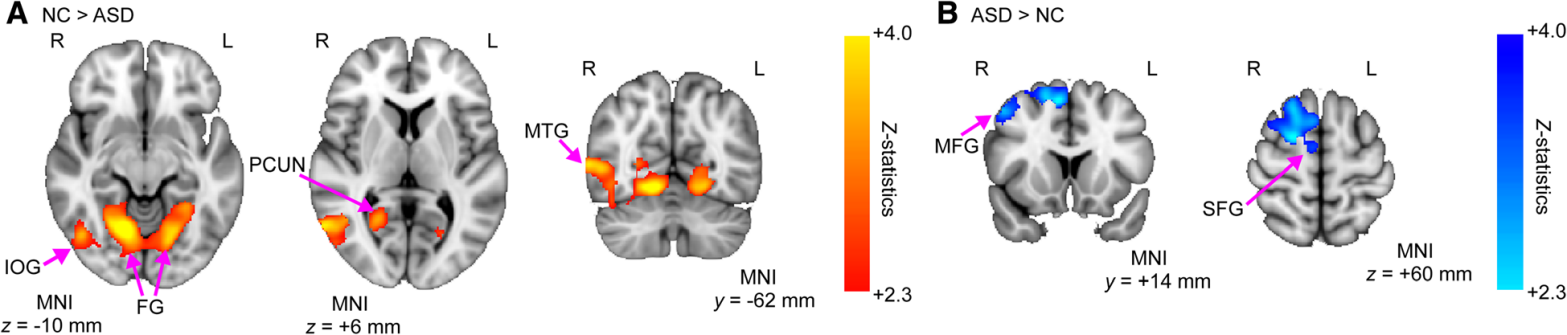
Atypical local connectivity in individuals with autism spectrum disorder (ASD) compared to normal controls (NCs). The *z*-maps of the group comparison of local connectivity are shown (NC>ASD and ASD>NC). Multiple comparisons were corrected based on the Gaussian random field theory (*z* > 2.3; cluster-level significance *p* < 0.05, corrected). The red-yellow color represents positive *z*-statistics, which indicates significant reductions in the group of individuals with ASD compared to the group of NCs. On the other hand, the light-blue color represents negative *z*-statistics, which indicates significant increases in the ASD group compared to the NC group. **a** The ASD group exhibited reduced local connectivity in a large cluster (4360 voxels) that included the bilateral LING, bilateral FG, and right MTG. **b** The ASD group showed enhanced local connectivity in a cluster (1809 voxels) that consisted of the right SFG, MFG, and PreCG. Right (*R*), left (*L*), lingual gyrus (*LING*), fusiform gyrus (*FG*), middle temporal gyrus (*MTG*), superior frontal gyrus (*SFG*), middle frontal gyrus (*MFG*), and precentral gyrus (*PreCG*)

Statistical analysis on distant connectivity revealed no clusters of voxels that showed a significant difference between the groups. In contrast, significant alterations in local connectivity were found in two clusters (Fig. 1 and Table 2), one of which included the bilateral fusiform gyri (FG), lingual gyri (LING), parahippocampal gyri (PHG), right inferior occipital gyrus (IOG), and MTG (Fig. 1a). In this cluster, participants with ASD exhibited significant decreases of local connectivity when compared to NCs. In the other cluster consisting of the right SFG, MFG, and precentral gyrus (PreCG), participants with ASD exhibited significant local over-connectivity relative to NCs (Fig. 1b).

**Table 2:** Altered local connectivity in participants with autism spectrum disorder relative to normal controls

Effect	Cluster size	MNI coordinate			Brain region	*z*-statistics
		*x*	*y*	*z*		
NC>ASD						
	4360	14	−62	−10	Lingual gyrus	4.31
					Fusiform gyrus	
					Posterior cingulate	
					Parahippocampus	
		60	−60	6	Middle temporal gyrus	3.94
					Inferior temporal gyrus	
					Superior temporal gyrus	
		−18	−66	−10	Lingual gyrus	3.78
					Fusiform gyrus	
					Parahippocampus	
		40	−52	−26	Cerebellum	3.18
		8	−74	20	Cuneus	3.14
ASD>NC						
	1809	20	0	60	Superior frontal gyrus	4.14
					Supplementary motor area	
		48	14	48	Middle frontal gyrus	3.54
		56	6	42	Precentral gyrus	4.14
					Inferior frontal gyrus	

### Fractional amplitude of low-frequency fluctuation

**Fig. 2:**
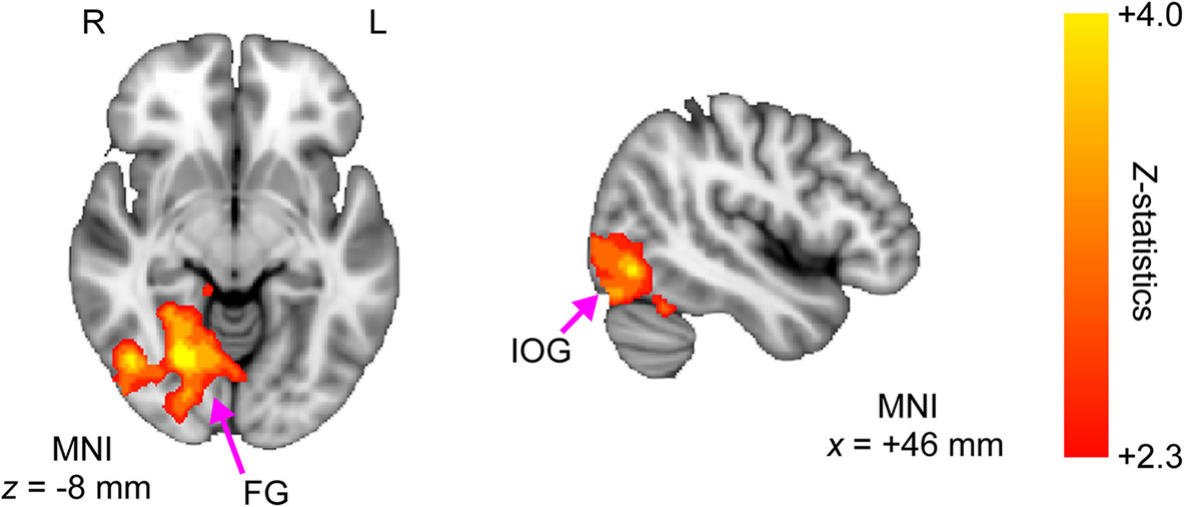
Reduced fractional amplitude of low-frequency fluctuation in individuals with autism spectrum disorder (ASD) compared with normal controls (NCs). The *z*-map of group comparisons of the fractional amplitude of low-frequency fluctuation (fALFF) is shown (NC>ASD). Multiple comparisons were corrected based on the Gaussian random field theory (*z* > 2.3; cluster-level significance *p* < 0.05, corrected). The red-yellow color represents positive *z*-statistics, which indicate significant reductions in the group of individuals with ASD compared to the group of NCs. Relative to the NC group, the ASD group exhibited significant decreases in fALFF in a large cluster (4437 voxels), including the right LING, FG, IOG, and PHG. Right (*R*), left (*L*), lingual gyrus (*LING*), fusiform gyrus (*FG*), inferior occipital gyrus (*IOG*), and parahippocampal gyrus (*PHG*)

Group comparisons revealed that participants with ASD showed significantly decreased fALFF values in a large cluster, including the right LING, middle occipital gyrus (MOG), FG, and cerebellum (Fig. 2 and Table 3). In contrast to the pattern of reduced local connectivity, no significant between-group differences were observed in the left hemisphere. However, the spatial pattern of reduced fALFF in the right hemisphere was similar to that of reduced local connectivity involving the posterior brain regions of the FG, IOG, and LING (c.f. Fig. 1a).

**Table 3:** Reduced fractional amplitude of low-frequency fluctuation in participants with autism spectrum disorder relative to normal controls

Effect	Cluster size	MNI coordinates			Brain region	*z*-statistics
		*x*	*y*	*z*		
NC>ASD						
	4437	22	−66	−8	Lingual gyrus	4.40
					Parahippocampus	
		46	−68	−8	Middle occipital gyrus	3.74
					Fusiform gyrus	
		−4	−86	2	Cuneus	3.24
		42	−54	−24	Fusiform gyrus	3.23
					Cerebellum crus 1	
					Culmen	

### Conjunction and spatial overlap analyses

**Fig. 3:**
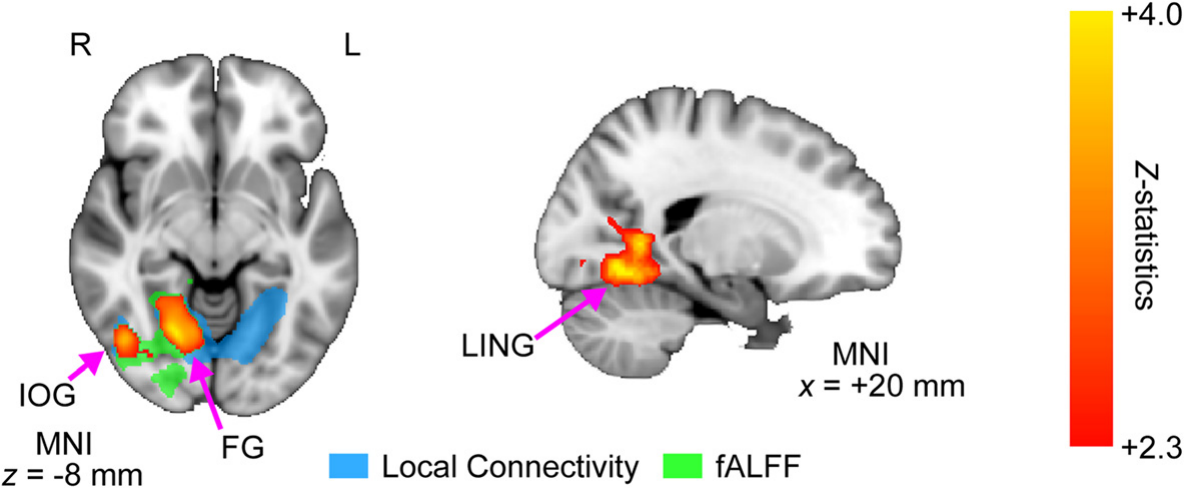
Spatial overlap of voxels showing alterations in local connectivity and fractional amplitude of low-frequency fluctuation in autism spectrum disorder (ASD). The *z*-map reflects the conjunction analysis between reduced local connectivity and reduced fractional amplitude of low-frequency fluctuation (fALFF). This analysis was performed using a cluster-wise test with *z*-stats threshold of 2.3 (corrected *p* threshold of 0.05). The red-yellow color indicates positive *z*-statistics (i.e., NC>ASD). In order to show the precise location of significant spatial overlap within the regions showing alterations of either measure, we overlaid the regions with disrupted local connectivity (shown in blue) and those showing reduced fALFF (shown in green) in the same axial slice. This analysis revealed substantial spatial overlaps between reductions in both local connectivity and fALFF. These significant spatial overlaps were observed in a large cluster (2047 voxels), which encompassed the right FG, LING, PCC, PHG, ITG, and pMTG. Right (*R*), left (*L*), fusiform gyrus (*FG*), lingual gyrus (*LING*), posterior cingulate cortex (*PCC*), parahippocampal gyrus (*PHG*), inferior occipital gyrus (*IOG*), inferior temporal gyrus (*ITG*), and posterior middle temporal gyrus (*pMTG*)

In order to evaluate the spatial overlap between alterations in local connectivity and those in fALFF more quantitatively, conjunction and spatial overlap analyses were performed. Conjunction analysis revealed that reductions of the two measures for participants with ASD clearly co-occurred in the right IOG, pMTG, LING, and FG (Fig. 3). In addition, the spatial overlaps between reductions in the two measures were further assessed using a Monte Carlo simulation with 5000 iterations [51, 52]. The simulation calculated the possibility of finding a larger number of overlapped voxels between the two measures by chance than that observed in the actual data (2047 voxels). According to the simulation, such probability was estimated as *p* = 0.0014, indicating that the spatial overlaps were unlikely to occur by chance.

### Relationships with autistic traits

**Fig. 4:**
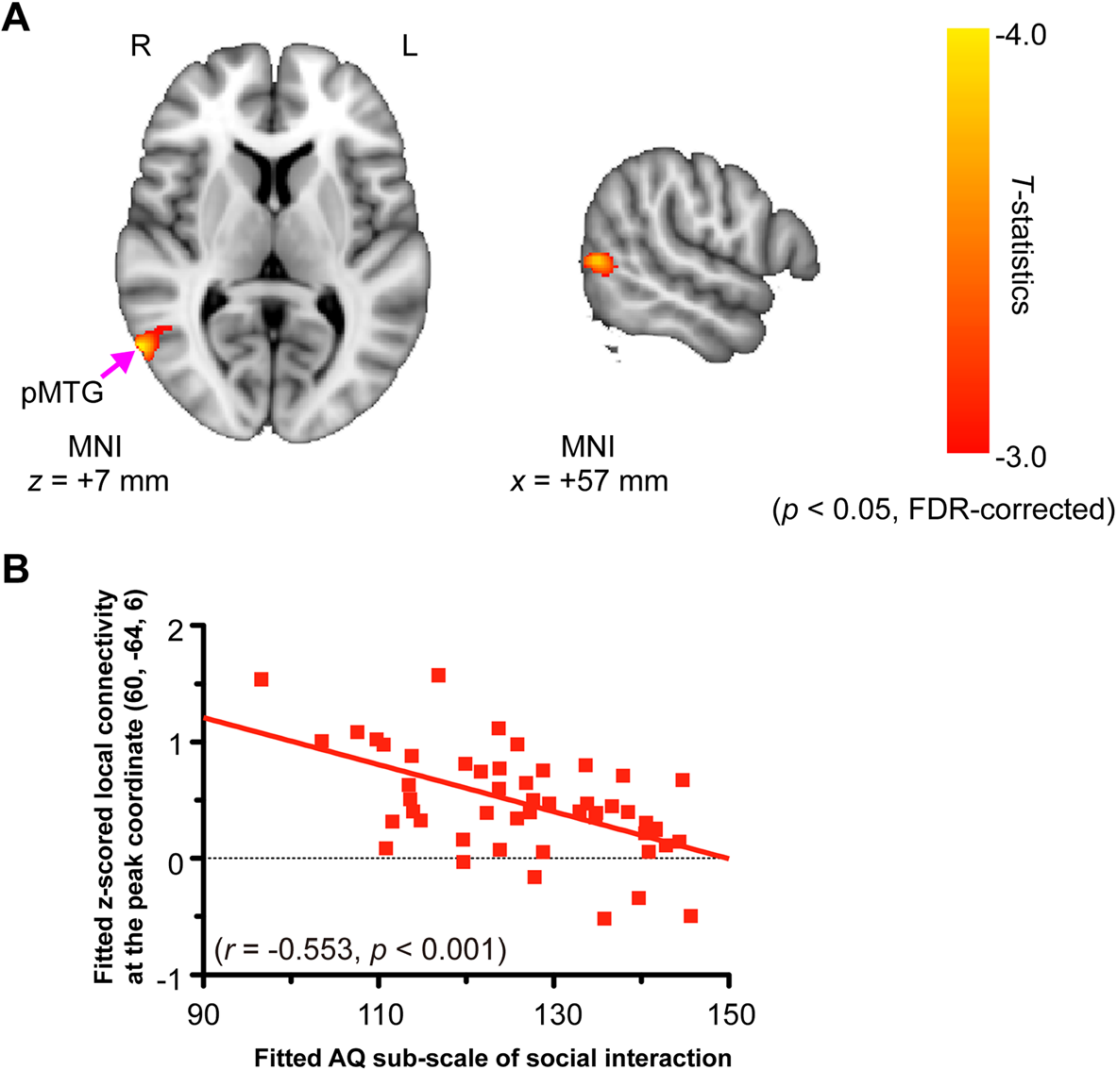
Significant negative correlations between disrupted local connectivity and the AQ subscale score of “social interaction”. A multiple linear regression analysis was performed in order to assess the relationship between disrupted local connectivity and the AQ “score of social interaction” subscale within the group of participants with autism spectrum disorder (ASD). **a** The multiple linear regression analysis revealed that the right pMTG showed a significant association between the AQ subscale score of “social interaction” and disrupted local connectivity. Of note, age and gender were included as nuisance covariates. Statistical significance was set at *p* < 0.05, with the false discovery rate (FDR) corrected for multiple comparisons. The red-yellow color indicates the negative *t*-statistics representing negative correlation between the AQ subscale of “social interaction” and local connectivity. **b** The relationship between disrupted local connectivity and the AQ subscale score of “social interaction” at the peak coordinate (MNI = [60, −64, 6]) was shown. For each participant with ASD, the local connectivity value was extracted from the peak coordinate and plotted against the “social interaction” score of that participant. Right (*R*), left (*L*), and posterior middle temporal gyrus (*pMTG*)

To examine whether these reductions in local connectivity and fALFF in localized brain regions have any relationships with the autistic traits, we performed multiple linear regression analysis using each AQ score (i.e., the total AQ score and the “social interaction” and “attention to detail” subscale scores) as an independent variable. Within the ASD group, no voxels showed significant associations of fALFF reductions with any of the AQ scores, including the total and subscale scores. In contrast, disrupted local connectivity in the right posterior MTG (pMTG) exhibited a highly significant negative correlation with the AQ subscale score of “social interaction” (Fig. 4).

## Discussion

In the present study, multiple aspects of spontaneous brain activity during rest were examined in adults with ASD using local connectivity, distant connectivity, and fALFF. Compared to the NC group, the ASD group showed significant alterations in local connectivity and fALFF but not in distant connectivity. The ASD group showed significant decreases of local connectivity in several occipital and temporal regions, including the bilateral FG, LING, and right pMTG together with increases of local connectivity in the right SFG and MFG. Furthermore, the ASD group exhibited significant reductions of fALFF values in regions in the occipital and temporal cortices, such as the right MOG, LING, and FG. Conjunction and spatial overlap analyses confirmed that the spatial pattern of reduced fALFF values substantially overlapped with that of reduced local connectivity particularly in the right IOG, pMTG, FG, and LING. Furthermore, within the ASD group, disrupted local connectivity in the right pMTG was significantly associated with the AQ subscale score of “social interaction”. These findings suggest that, in adults with ASD, local functional disruptions in several social brain regions are manifested in the form of co-occurred alterations in the magnitude and local connectivity during spontaneous brain activity and that such local functional disruptions may underlie core problems of their social and communicative difficulties.

### Atypical local connectivity in the ASD brain

A number of previous rsfMRI studies have demonstrated that individuals with ASD show atypical functional connectivity among several brain regions [8, 16, 24, 30, 31, 53–55]. The majority of studies that examined adults with ASD have found largely reduced connectivity patterns [3]. Although children with ASD often show over-connectivity [13, 31, 56, 57], only a few studies have reported over-connectivity in adults [12]. However, it is important to note that previous rsfMRI studies on adults with ASD focused on intrinsic connectivity in a relatively large spatial scale such as connectivity between brain regions defined on the level of gyri or by interaction between functional networks (i.e., functional network connectivity), thus leaving possible alterations in local functional connectivity unexplored. To the best of our knowledge, the present rsfMRI study is the first that has demonstrated both under- and over-connectivity patterns in the localized brain regions in adult ASD using the same analytical framework for characterizing distant connectivity.

In this study, local over-connectivity was found in the right SFG and MFG. Behaviorally, individuals with ASD often show enhanced perceptual functioning particularly in the visual domain, as evidenced by superior performance in tasks that require extensive local visual information processing such as visual search [58–61]. These observations led to a proposal that visual perception of ASD is more locally oriented than that of typical individuals [62]. This view raises a possibility that such preference in ASD might be linked with atypical brain activity in brain regions related to visual information processing and its control. Indeed, previous fMRI studies have demonstrated that the SFG is critically involved in visuospatial attention and visual spatial working memory [63–66], and that individuals with ASD show atypical activation in the SFG while attending to local patterns in the hierarchical shape-recognition task [67]. Considering these findings, our results may suggest a possibility that local over-connectivity in the right SFG and MFG might be related to atypical visual information processing in ASD.

In addition to local over-connectivity in the frontal regions, the ASD group exhibited local under-connectivity in the occipital and temporal regions, including the bilateral FG, LING, and right pMTG. These regions have been known to play crucial roles in various social and communicative functions, including face perception [68–70], identification of facial expression of emotion [71], and detection and processing of social cues (e.g., gaze) [72, 73], all of which are disrupted in ASD [74, 75]. In contrast to our findings in adults, Keown et al. [24] and Maximo et al. [23] recently observed that adolescents with ASD showed local over-connectivity in the occipital and temporal regions. Here, it is noteworthy that individuals with ASD have been shown to follow an atypical course of developmental changes in intrinsic connectivity from late childhood to early adulthood, showing both age-related increase and decrease depending on brain regions [2]. Thus, it is possible to postulate that discrepancy in findings between adults and adolescents with ASD may reflect maturational changes from puberty to adulthood. Such a possibility may be addressed by future longitudinal studies.

We also found a significant association between disrupted local connectivity in the right pMTG and the social interaction deficits as assessed by the AQ. In line with previous findings, individuals with ASD have shown atypical brain activation in this region during eye-gaze processing [75, 76]. Furthermore, Nummenmaa et al. [77] demonstrated that the neural response to eye-gaze processing (variable versus constant eye-gaze) in this region was significantly associated with the total AQ score in the neurotypical population. Taken together, our findings suggest that disrupted local connectivity in the right pMTG may be particularly linked to atypical gaze processing, which may contribute to socio-communicative deficits in individuals with ASD.

### Atypical spontaneous brain activity level in ASD

The fALFF measures the amplitude of spontaneous blood-oxygen-level-dependent (BOLD) fluctuations [78]. Previous studies have demonstrated coupling between BOLD activity and local field potential in anesthetized monkey [79] and human [80]. Furthermore, a recent study has acquired high-resolution fMRI and electrocorticography (ECoG) data from the same human subjects and has revealed that the spatial pattern of BOLD activation matches that of ECoG power changes in the sensorimotor cortex [81]. Considering these findings, significant changes in fALFF may reflect a portion of abnormal electrophysiological activity in local brain region.

Although the importance of the relationship between altered spontaneous brain activity and ASD etiology has been widely acknowledged, the majority of previous rsfMRI studies of ASD have focused on functional connectivity and only a few have examined spontaneous brain activity in this disorder [30, 31]. In the present study, we found that participants with ASD exhibited significant fALFF reductions in multiple posterior brain regions, including the right MOG, FG, LING, and cerebellum. Given accumulating evidence from simultaneous recording data showing a tight correspondence between BOLD and electrophysiological signals in human and animal subjects [79–81], significant changes in the amplitudes of BOLD fluctuations as measured by fALFF are likely to reflect a portion of abnormal electrophysiological activity in these localized brain regions. Yizhar et al. [82] have suggested that alterations in the cellular balance of excitation and inhibition within neural microcircuitry may cause social and cognitive dysfunctions in ASD. Taking account of these findings, we postulate that the current results might reflect aberrant neuronal activity induced by the atypical underlying microcircuitry in the ASD brain.

### Co-occurrence of disrupted local connectivity and reduced brain activity level in ASD

Conjunction and spatial analyses have revealed that in addition to the right pMTG, the ASD group shows co-occurred reductions of local functional connectivity and spontaneous activity level in the FG. Morphological and functional anomalies of the FG in ASD have been reported in a number of previous studies. For instance, a *postmortem* study has revealed significant reductions in neuron densities in layer III, total number of neurons in layers (III, V, and IV), and the mean perikaryal volumes of neurons in layers (V and VI) in the FG [83]. In addition, positron emission tomography (PET) studies have demonstrated that adults with ASD show atypical acetylcholinesterase and microglial activations in this region [84, 85]. Moreover, a number of fMRI studies have reported that individuals with ASD show hypo-activity during face perception [86]. All these findings indicate disrupted local organization and activity in the FG. Notably, a recent study using simultaneous recording of PET and fMRI has demonstrated that local neuronal activity determines intrinsic BOLD connectivity during rest in the human brain [87]. A causal relationship between reduction of local connectivity and level of brain activity in the FG remains unclear. However, simultaneous alterations in the two key functional measures of spontaneous brain activity in the FG indicate that, in addition to the right pMTG, local functional disruption of this region would be a key factor that contributes to the core clinical symptoms of ASD.

### Limitations and considerations

We discuss here several caveats of the present study. First, we determined the radii for distinguishing between local and distant connectivity based on criteria adopted by a previous study [48]: local connectivity was defined as the weighted degree of connectivity within a 12-mm-radius sphere, while distant connectivity was defined as that outside a 25-mm-radius sphere. However, current literature has not reached unequivocal criteria for determining these two types of connectivity. For instance, local connectivity has been defined by using either a 12- [41, 48, 88] or a 14-mm-radius [4, 22–24] sphere, and distant connectivity has also been defined by using a 12- [88], 14- [4, 22], or a 25-mm-radius sphere [48]. Compared with distant connectivity, the number of voxels included in calculating local connectivity is substantially smaller, which may lead one to wonder whether even a small difference in radius may result in a drastic change for local connectivity. However, Sepulcre et al. [22] have examined the effects of radius on estimation of local connectivity and have confirmed no significant changes on the spatial patterns of local connectivity if the radius lies within the range between 10 and 14 mm. We did examine local and distant connectivity in multiple spatial scales (local connectivity 10- and 14-mm radii; distant connectivity 23- and 27-mm radii), in order to confirm whether our findings in local connectivity was not due to arbitrary definitions of local and distant connectivity. For local connectivity, significant reductions for ASD in the bilateral FG, LING, and right MTG, accompanied by a significant increase in the right SFG, were consistent with the main analysis using the 12-mm radius (see Fig. 1 and Additional file 1: Figure S1). Also consistent with the main analysis using the 25-mm radius, we found no significant between-group difference in distant connectivity in any spatial scale. Therefore, our major findings are robust to a range of choices of radii determining local and distant connectivity.

Second, contrary to our initial hypothesis, we observed no significant differences in distant connectivity between the two groups. This counter-intuitive result may be due to the difference in the method for calculating distant connectivity from the majority of previous studies: while connectivity is conventionally estimated by calculating temporal correlation between two specific brain regions or voxels, distant connectivity in the present study was computed as the sum of all the weighted edges (or voxel-to-voxel connections) outside of a 25-mm-radius sphere centering a focal node (around 144,170 edges or connections). In this method, there is a possibility that, even though region-specific under and over-connectivity exist in the ASD brain, the summation with numerous other normal connections and altered connections with the opposite direction may mask the effect of such region-specific abnormalities. In this study, we nonetheless adopted this approach because it has an advantage over other region-of-interest (ROI)-based and seed-based analyses in that it does not require specific assumptions or hypotheses regarding the choice of paired ROIs or seed region to examine the strength of functional coupling between distant regions. In addition, the method allowed us to estimate both local and distant connectivity under the same analytical framework. Furthermore, several lines of evidence suggest that functional abnormalities in the ASD brain are widely distributed [6, 30], making it difficult to specify a pair of ROIs or seeds in advance. Therefore, we thought that this method might be suitable to examine potential alterations in distant connectivity in the ASD brain.

Third, the ASD group in this study was composed of patients with three ASD subtypes: high-functioning autism (HFA; *n* = 22), Asperger’s syndrome (AS; *n* = 17), and pervasive developmental disorder not otherwise specified (PDD-NOS; *n* = 11). Differences in the behavior and neuroimaging measures among the ASD subtypes have been highly controversial, with some studies reporting positive results [89, 90] and others reporting negative ones [91]. Therefore, it would be interesting to know whether and how these ASD subtypes differ in local connectivity and fALFF. To examine this possibility, we performed a one-way analysis of variance (ANOVA) on each measure of local connectivity and fALFF with group (the three ASD subtypes) as a factor. Each measure was extracted from a 5-mm-radius sphere centered at the coordinate (MNI = [20, −64, −12]) with the peak *z*-statistics obtained by conjunction analysis. We found that the main effect of the group was not close to significance either for local connectivity (*F* = 0.37, *p* = 0.69) or for fALFF (*F* = 0.29, *p* = 0.75). Although a larger sample size and a more systematic analysis are needed to draw firm conclusions regarding the possible differences among the ASD subtypes, the absence of significant main effects for ASD subtype indicated that the present results were not driven only by a certain subtype of ASD.

Fourth, we observed reduced fALFF only in the right occipital regions and no enhanced fALFF in the ASD group. However, a previous study revealed increased fALFF in the right frontal regions and decreases of this measure in the left occipital regions [30]. Because the previous study was a multicenter large cohort study that included childhood data, the discrepancies in the results might be due to many factors, including those related to the population and variable scanning conditions. Because the number of studies is limited, further investigations are needed to establish the pattern of alterations of fALFF in adults with ASD.

Fifth, it is noteworthy that the cluster of voxels showing significant correlation with the AQ score (“social interaction” subscale) was found outside the overlaps between the regions with reduced local connectivity and those with reduced fALFF. Within the overlapping voxels between the two maps, we did not find any voxels showing significant correlation of any AQ score with either local connectivity or fALFF, even with a more liberal threshold (*p* < 0.01, uncorrected for multiple comparisons). It is possible that the converging zones of the two altered physiological processes may reflect some traits and/or states, such as altered sensory perception and cognition, which are widely observed in the adult ASD population but which are not particularly evaluated by the AQ. Future studies that employ more specialized questionnaires and behavioral assessments may help to clarify the functional significance of the overlapping regions for behavioral problems in ASD.

## Conclusions

In conclusion, the present study examined two different aspects of spontaneous brain activity of the ASD brain using graph-theoretic and frequency-domain analyses. We observed that reductions in the local connectivity and in the amplitude of low frequency fluctuation co-occurred in the right FG and pMTG, regions critically involved in processing social signals such as face and eye gaze and in other crucial social functions. In particular, disrupted local connectivity in the right pMTG was significantly associated with the “social interaction” subscale of the AQ. These findings indicate that functional disruptions of local neural circuitry in these social brain regions may underlie social and communicative dysfunctions in adult ASD. Our approach of investigating multiple aspects of spontaneous brain activity holds the promise of leading to a unified understanding of crucial pathophysiological processes affecting the ASD brain.

## Additional file

Additional file 1: Figure S1. Group comparisons of local connectivity in multiple spatial scales. In addition to the main analysis (12-mm radius), local connectivity was examined in multiple spatial scales (10- and 14-mm radii). For the main analysis, see Fig. 1. The *z*-maps of the group comparison are shown (NC>ASD in the red-yellow color and ASD>NC in the light blue color). Statistical threshold was set at *p* < 0.05, corrected for multiple comparisons at the cluster-level. Note that significant reductions for ASD in the bilateral FG, LING, and the right MTG, together with a significant increase in the right SFG, were replicated in all the three conditions.

## Abbreviations

AFNI: Analysis of Functional NeuroImages
ALFF: amplitude of low-frequency fluctuation
ANOVA: analysis of variance
AQ: autism-spectrum quotient
ASD: autism spectrum disorder
BOLD: blood oxygen level-dependent
DSM: Diagnostic and Statistical Manual of Mental Disorders
DVARS: D referring temporal derivative and VARS referring the root mean squared
ECoG: electrocorticography
fALFF: fractional amplitude of low-frequency fluctuation
FD: frame-wise displacement
FDR: false discovery rate
FG: Fusiform gyrus
fMRI: functional magnetic resonance imaging
FMRIB: functional MRI of the brain
FSL: FMRIB’s Software Library
IOG: inferior occipital gyrus
IQ: intelligence quotient
JART: Japanese version of the National Adult Reading Test
KCC: Kendall’s coefficient of concordance
LING: lingual gyrus
MFG: middle frontal gyrus
MNI: Montreal Neurological Institute
MOG: middle occipital gyrus
MTG: middle temporal gyrus
NC: normal control
PCC: posterior cingulate cortex
PET: positron emission tomography
PHG: parahippocampal gyrus
pMTG: posterior middle temporal gyrus
PreCG: precentral gyrus
ReHo: regional homogeneity
ROI: region of interest
rsfMRI: resting-state functional magnetic resonance imaging
SFG: superior frontal gyrus
SPGR: spoiled gradient recalled
SPM: Statistical Parametric Mapping
STG: superior temporal gyrus
TE: echo time
TR: repetition time
WAIS: Wechsler Adult Intelligence Scale

## References

[1] Vissers ME, Cohen MX, Geurts HM (2012). Brain connectivity and high functioning autism: a promising path of research that needs refined models, methodological convergence, and stronger behavioral links. Neurosci Biobehav Rev.

[2] Padmanabhan A, Lynn A, Foran W, Luna B, O’Hearn K (2013). Age related changes in striatal resting state functional connectivity in autism. Front Hum Neurosci..

[3] Uddin LQ, Supekar K, Menon V (2013). Reconceptualizing functional brain connectivity in autism from a developmental perspective. Front Hum Neurosci..

[4] You X, Norr M, Murphy E, Kuschner ES, Bal E, Gaillard WD (2013). Atypical modulation of distant functional connectivity by cognitive state in children with autism spectrum disorders. Front Hum Neurosci..

[5] Just MA, Cherkassky VL, Keller TA, Minshew NJ (2004). Cortical activation and synchronization during sentence comprehension in high-functioning autism: evidence of underconnectivity. Brain.

[6] Muller RA (2007). The study of autism as a distributed disorder. Ment Retard Dev Disabil Res Rev.

[7] Just MA, Cherkassky VL, Keller TA, Kana RK, Minshew NJ (2007). Functional and anatomical cortical underconnectivity in autism: evidence from an FMRI study of an executive function task and corpus callosum morphometry. Cereb Cortex.

[8] Assaf M, Jagannathan K, Calhoun VD, Miller L, Stevens MC, Sahl R (2010). Abnormal functional connectivity of default mode sub-networks in autism spectrum disorder patients. Neuroimage.

[9] Kennedy DP, Redcay E, Courchesne E (2006). Failing to deactivate: resting functional abnormalities in autism. Proc Natl Acad Sci U S A.

[10] Kana RK, Keller TA, Cherkassky VL, Minshew NJ, Just MA (2006). Sentence comprehension in autism: thinking in pictures with decreased functional connectivity. Brain.

[11] Di Martino A, Kelly C, Grzadzinski R, Zuo XN, Mennes M, Mairena MA (2011). Aberrant striatal functional connectivity in children with autism. Biol Psychiatry.

[12] Monk CS, Peltier SJ, Wiggins JL, Weng SJ, Carrasco M, Risi S (2009). Abnormalities of intrinsic functional connectivity in autism spectrum disorders. Neuroimage.

[13] Delmonte S, Gallagher L, O’Hanlon E, McGrath J, Balsters JH (2013). Functional and structural connectivity of frontostriatal circuitry in autism spectrum disorder. Front Hum Neurosci..

[14] Noonan SK, Haist F, Muller RA (2009). Aberrant functional connectivity in autism: evidence from low-frequency BOLD signal fluctuations. Brain Res..

[15] Kana RK, Libero LE, Moore MS (2011). Disrupted cortical connectivity theory as an explanatory model for autism spectrum disorders. Phys Life Rev.

[16] Wiggins JL, Peltier SJ, Ashinoff S, Weng SJ, Carrasco M, Welsh RC (2011). Using a self-organizing map algorithm to detect age-related changes in functional connectivity during rest in autism spectrum disorders. Brain Res..

[17] Vargas DL, Nascimbene C, Krishnan C, Zimmerman AW, Pardo CA (2005). Neuroglial activation and neuroinflammation in the brain of patients with autism. Ann Neurol.

[18] Courchesne E, Pierce K (2005). Why the frontal cortex in autism might be talking only to itself: local over-connectivity but long-distance disconnection. Curr Opin Neurobiol.

[19] Zang Y, Jiang T, Lu Y, He Y, Tian L (2004). Regional homogeneity approach to fMRI data analysis. Neuroimage.

[20] Paakki JJ, Rahko J, Long X, Moilanen I, Tervonen O, Nikkinen J (2010). Alterations in regional homogeneity of resting-state brain activity in autism spectrum disorders. Brain Res..

[21] Shukla DK, Keehn B, Muller RA (2010). Regional homogeneity of fMRI time series in autism spectrum disorders. Neurosci Lett.

[22] Sepulcre J, Liu H, Talukdar T, Martincorena I, Yeo BT, Buckner RL (2010). The organization of local and distant functional connectivity in the human brain. PLoS Comput Biol.

[23] Maximo JO, Keown CL, Nair A, Muller RA (2013). Approaches to local connectivity in autism using resting state functional connectivity MRI. Front Hum Neurosci..

[24] Keown CL, Shih P, Nair A, Peterson N, Mulvey ME, Muller RA (2013). Local functional overconnectivity in posterior brain regions is associated with symptom severity in autism spectrum disorders. Cell Rep.

[25] Biswal BB, Mennes M, Zuo XN, Gohel S, Kelly C, Smith SM (2010). Toward discovery science of human brain function. Proc Natl Acad Sci U S A.

[26] Han Y, Wang J, Zhao Z, Min B, Lu J, Li K (2011). Frequency-dependent changes in the amplitude of low-frequency fluctuations in amnestic mild cognitive impairment: a resting-state fMRI study. Neuroimage.

[27] Liu Y, Yu C, Zhang X, Liu J, Duan Y, Alexander-Bloch AF (2013). Impaired long distance functional connectivity and weighted network architecture in Alzheimer’s disease. Cerebral Cortex..

[28] Wang L, Dai W, Su Y, Wang G, Tan Y, Jin Z (2012). Amplitude of low-frequency oscillations in first-episode, treatment-naive patients with major depressive disorder: a resting-state functional MRI study. PLoS One.

[29] Zang YF, He Y, Zhu CZ, Cao QJ, Sui MQ, Liang M (2007). Altered baseline brain activity in children with ADHD revealed by resting-state functional MRI. Brain Dev.

[30] Di Martino A, Yan CG, Li Q, Denio E, Castellanos FX, Alaerts K (2013). The autism brain imaging data exchange: towards a large-scale evaluation of the intrinsic brain architecture in autism. Mol Psychiatry..

[31] Supekar K, Uddin LQ, Khouzam A, Phillips J, Gaillard WD, Kenworthy LE (2013). Brain hyperconnectivity in children with autism and its links to social deficits. Cell Rep.

[32] Bos DJ, van Raalten TR, Oranje B, Smits AR, Kobussen NA, Belle J (2014). Developmental differences in higher-order resting-state networks in autism spectrum disorder. Neuroimage Clin..

[33] Matsuoka K, Uno M, Kasai K, Koyama K, Kim Y (2006). Estimation of premorbid IQ in individuals with Alzheimer’s disease using Japanese ideographic script (Kanji) compound words: Japanese version of national adult reading test. Psychiatry Clin Neurosci.

[34] Wakabayashi A, Baron-Cohen S, Wheelwright S, Tojo Y (2006). The autism-spectrum quotient (AQ) in Japan: a cross-cultural comparison. J Autism Dev Disord.

[35] Hoekstra RA, Bartels M, Cath DC, Boomsma DI (2008). Factor structure, reliability and criterion validity of the autism-spectrum quotient (AQ): a study in Dutch population and patient groups. J Autism Dev Disord.

[36] Cox RW (1996). AFNI: software for analysis and visualization of functional magnetic resonance neuroimages. Comput Biomed Res.

[37] Smith SM, Jenkinson M, Woolrich MW, Beckmann CF, Behrens TE, Johansen-Berg H (2004). Advances in functional and structural MR image analysis and implementation as FSL. Neuroimage..

[38] Behzadi Y, Restom K, Liau J, Liu TT (2007). A component based noise correction method (CompCor) for BOLD and perfusion based fMRI. Neuroimage.

[39] Murphy K, Birn RM, Handwerker DA, Jones TB, Bandettini PA (2009). The impact of global signal regression on resting state correlations: are anti-correlated networks introduced?. Neuroimage.

[40] Power JD, Barnes KA, Snyder AZ, Schlaggar BL, Petersen SE (2012). Spurious but systematic correlations in functional connectivity MRI networks arise from subject motion. Neuroimage.

[41] Van Dijk KR, Sabuncu MR, Buckner RL (2012). The influence of head motion on intrinsic functional connectivity MRI. Neuroimage.

[42] Carp J (2013). Optimizing the order of operations for movement scrubbing: comment on Power et al. Neuroimage..

[43] Di Martino A, Zuo XN, Kelly C, Grzadzinski R, Mennes M, Schvarcz A (2013). Shared and distinct intrinsic functional network centrality in autism and attention-deficit/hyperactivity disorder. Biol Psychiatry.

[44] Zuo XN, Ehmke R, Mennes M, Imperati D, Castellanos FX, Sporns O (2012). Network centrality in the human functional connectome. Cereb Cortex.

[45] Buckner RL, Sepulcre J, Talukdar T, Krienen FM, Liu H, Hedden T (2009). Cortical hubs revealed by intrinsic functional connectivity: mapping, assessment of stability, and relation to Alzheimer’s disease. J Neurosci.

[46] Hwang K, Hallquist MN, Luna B (2013). The development of hub architecture in the human functional brain network. Cereb Cortex.

[47] Hagmann P, Cammoun L, Gigandet X, Meuli R, Honey CJ, Wedeen VJ (2008). Mapping the structural core of human cerebral cortex. PLoS Biol.

[48] Mueller S, Wang D, Fox MD, Yeo BT, Sepulcre J, Sabuncu MR (2013). Individual variability in functional connectivity architecture of the human brain. Neuron.

[49] Song XW, Dong ZY, Long XY, Li SF, Zuo XN, Zhu CZ (2011). REST: a toolkit for resting-state functional magnetic resonance imaging data processing. PLoS One.

[50] Nichols T, Brett M, Andersson J, Wager T, Poline JB (2005). Valid conjunction inference with the minimum statistic. Neuroimage.

[51] Lombardo MV, Ashwin E, Auyeung B, Chakrabarti B, Taylor K, Hackett G (2012). Fetal testosterone influences sexually dimorphic gray matter in the human brain. J Neurosci.

[52] Lai MC, Lombardo MV, Suckling J, Ruigrok AN, Chakrabarti B, Ecker C (2013). Biological sex affects the neurobiology of autism. Brain.

[53] Abrams DA, Lynch CJ, Cheng KM, Phillips J, Supekar K, Ryali S (2013). Underconnectivity between voice-selective cortex and reward circuitry in children with autism. Proc Natl Acad Sci.

[54] Ebisch SJ, Gallese V, Willems RM, Mantini D, Groen WB, Romani GL (2011). Altered intrinsic functional connectivity of anterior and posterior insula regions in high-functioning participants with autism spectrum disorder. Hum Brain Mapp.

[55] Kennedy DP, Courchesne E (2008). The intrinsic functional organization of the brain is altered in autism. Neuroimage.

[56] Uddin LQ, Supekar K, Lynch CJ, Khouzam A, Phillips J, Feinstein C (2013). Salience network-based classification and prediction of symptom severity in children with autism. JAMA Psychiatry.

[57] Lynch CJ, Uddin LQ, Supekar K, Khouzam A, Phillips J, Menon V (2013). Default mode network in childhood autism: posteromedial cortex heterogeneity and relationship with social deficits. Biol Psychiatry.

[58] Mottron L, Burack JA, Iarocci G, Belleville S, Enns JT (2003). Locally oriented perception with intact global processing among adolescents with high-functioning autism: evidence from multiple paradigms. J Child Psychol Psychiatry.

[59] O’Riordan MA, Plaisted KC, Driver J, Baron-Cohen S (2001). Superior visual search in autism. J Exp Psychol Hum Percept Perform.

[60] Muth A, Honekopp J, Falter CM (2014). Visuo-spatial performance in autism: a meta-analysis. J Autism Dev Disord.

[61] Blaser E, Eglington L, Carter AS, Kaldy Z (2014). Pupillometry reveals a mechanism for the autism spectrum disorder (ASD) advantage in visual tasks. Sci Rep..

[62] Mottron L, Dawson M, Soulieres I, Hubert B, Burack J (2006). Enhanced perceptual functioning in autism: an update, and eight principles of autistic perception. J Autism Dev Disord.

[63] Corbetta M, Kincade JM, Shulman GL (2002). Neural systems for visual orienting and their relationships to spatial working memory. J Cogn Neurosci.

[64] Liu Y, Bengson J, Huang H, Mangun GR, Ding M (2014). Top-down modulation of neural activity in anticipatory visual attention: control mechanisms revealed by simultaneous EEG-fMRI. Cereb Cortex.

[65] Hopfinger JB, Buonocore MH, Mangun GR (2000). The neural mechanisms of top-down attentional control. Nat Neurosci.

[66] Hahn B, Ross TJ, Stein EA (2006). Neuroanatomical dissociation between bottom-up and top-down processes of visuospatial selective attention. Neuroimage.

[67] Gadgil M, Peterson E, Tregellas J, Hepburn S, Rojas DC (2013). Differences in global and local level information processing in autism: an fMRI investigation. Psychiatry Res.

[68] Avidan G, Tanzer M, Hadj-Bouziane F, Liu N, Ungerleider LG, Behrmann M (2013). Selective dissociation between core and extended regions of the face processing network in congenital prosopagnosia. Cereb Cortex..

[69] Behrmann M, Plaut DC (2013). Distributed circuits, not circumscribed centers, mediate visual recognition. Trends Cogn Sci.

[70] Cohen Kadosh K, Johnson MH (2007). Developing a cortex specialized for face perception. Trends Cogn Sci.

[71] Kitada R, Johnsrude IS, Kochiyama T, Lederman SJ (2010). Brain networks involved in haptic and visual identification of facial expressions of emotion: an fMRI study. Neuroimage.

[72] Pelphrey KA, Morris JP, McCarthy G (2004). Grasping the intentions of others: the perceived intentionality of an action influences activity in the superior temporal sulcus during social perception. J Cogn Neurosci.

[73] Sugiura M, Yomogida Y, Mano Y, Sassa Y, Kambara T, Sekiguchi A (2013). From social-signal detection to higher social cognition: an fMRI approach. Social Cognitive and Affective Neuroscience..

[74] Deeley Q, Daly EM, Surguladze S, Page L, Toal F, Robertson D (2007). An event related functional magnetic resonance imaging study of facial emotion processing in Asperger syndrome. Biol Psychiatry.

[75] von dem Hagen EA, Stoyanova RS, Rowe JB, Baron-Cohen S, Calder AJ (2013). Direct gaze elicits atypical activation of the theory-of-mind network in autism spectrum conditions. Cereb Cortex..

[76] Georgescu AL, Kuzmanovic B, Schilbach L, Tepest R, Kulbida R, Bente G (2013). Neural correlates of “social gaze” processing in high-functioning autism under systematic variation of gaze duration. Neuroimage Clin..

[77] Nummenmaa L, Engell AD, von dem Hagen E, Henson RN, Calder AJ (2012). Autism spectrum traits predict the neural response to eye gaze in typical individuals. Neuroimage.

[78] Zou QH, Zhu CZ, Yang Y, Zuo XN, Long XY, Cao QJ (2008). An improved approach to detection of amplitude of low-frequency fluctuation (ALFF) for resting-state fMRI: fractional ALFF. J Neurosci Methods.

[79] Logothetis NK, Pauls J, Augath M, Trinath T, Oeltermann A (2001). Neurophysiological investigation of the basis of the fMRI signal. Nature.

[80] Mukamel R, Gelbard H, Arieli A, Hasson U, Fried I, Malach R (2005). Coupling between neuronal firing, field potentials, and FMRI in human auditory cortex. Science.

[81] Siero JC, Hermes D, Hoogduin H, Luijten PR, Ramsey NF, Petridou N (2014). BOLD matches neuronal activity at the mm scale: a combined 7T fMRI and ECoG study in human sensorimotor cortex. Neuroimage..

[82] Yizhar O, Fenno LE, Prigge M, Schneider F, Davidson TJ, O’Shea DJ (2011). Neocortical excitation/inhibition balance in information processing and social dysfunction. Nature.

[83] van Kooten IA, Palmen SJ, von Cappeln P, Steinbusch HW, Korr H, Heinsen H (2008). Neurons in the fusiform gyrus are fewer and smaller in autism. Brain.

[84] Suzuki K, Sugihara G, Ouchi Y, Nakamura K, Futatsubashi M, Takebayashi K (2013). Microglial activation in young adults with autism spectrum disorder. JAMA Psychiatry.

[85] Suzuki K, Sugihara G, Ouchi Y, Nakamura K, Tsujii M, Futatsubashi M (2011). Reduced acetylcholinesterase activity in the fusiform gyrus in adults with autism spectrum disorders. Arch Gen Psychiatry.

[86] Pierce K, Muller RA, Ambrose J, Allen G, Courchesne E (2001). Face processing occurs outside the fusiform ‘face area’ in autism: evidence from functional MRI. Brain.

[87] Riedl V, Bienkowska K, Strobel C, Tahmasian M, Grimmer T, Forster S (2014). Local activity determines functional connectivity in the resting human brain: a simultaneous FDG-PET/fMRI study. J Neurosci.

[88] Beucke JC, Sepulcre J, Talukdar T, Linnman C, Zschenderlein K, Endrass T (2013). Abnormally high degree connectivity of the orbitofrontal cortex in obsessive-compulsive disorder. JAMA Psychiatry.

[89] McAlonan GM, Cheung C, Cheung V, Wong N, Suckling J, Chua SE (2009). Differential effects on white-matter systems in high-functioning autism and Asperger’s syndrome. Psychol Med.

[90] McAlonan GM, Suckling J, Wong N, Cheung V, Lienenkaemper N, Cheung C (2008). Distinct patterns of grey matter abnormality in high-functioning autism and Asperger’s syndrome. J Child Psychol Psychiatry.

[91] Via E, Radua J, Cardoner N, Happe F, Mataix-Cols D (2011). Meta-analysis of gray matter abnormalities in autism spectrum disorder: should Asperger disorder be subsumed under a broader umbrella of autistic spectrum disorder?. Arch Gen Psychiatry.

