# Supplementary figures and images for: Alterations of local spontaneous brain activity and connectivity in adults with high-functioning autism spectrum disorder
Source: Mol Autism. 2015 May 24;6:30. doi: 10.1186/s13229-015-0026-z (PMC4446946; doi:10.1186/s13229-015-0026-z)

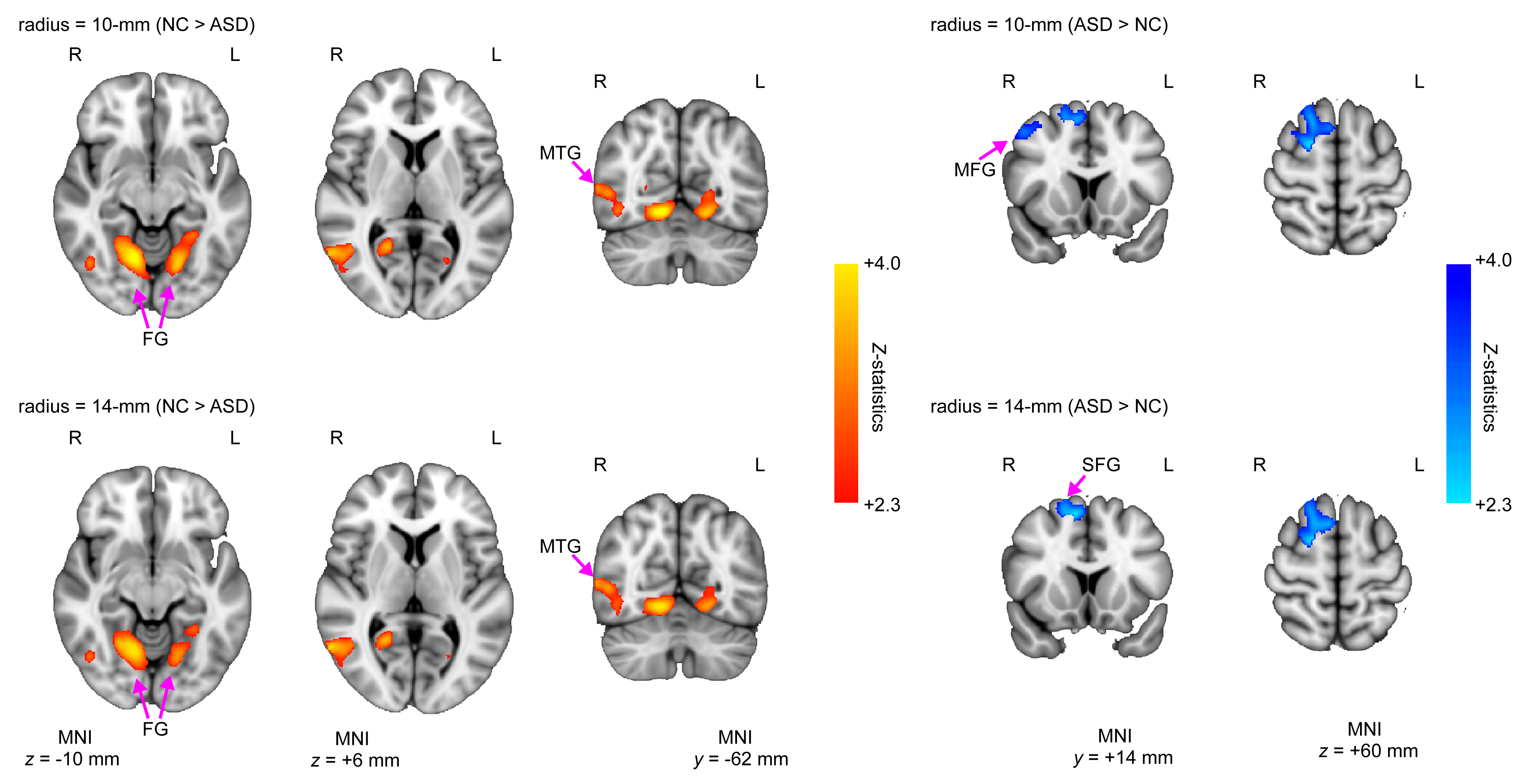

Supplement: Additional file 1: Figure S1. — Group comparisons of local connectivity in multiple spatial scales. In addition to the main analysis (12-mm radius), local connectivity was examined in multiple spatial scales (10- and 14-mm radii). For the main analysis, see Fig. 1. The z-maps of the group comparison are shown (NC>ASD in the red-yellow color and ASD>NC in the light blue color). Statistical threshold was set at p < 0.05, corrected for multiple comparisons at the cluster-level. Note that significant reductions for ASD in the bilateral FG, LING, and the right MTG, together with a significant increase in the right SFG, were replicated in all the three conditions. [file 13229_2015_26_MOESM1_ESM.tiff]
